# Supplementary material for: Perception and practice of self-medication with antibiotics among medical students in Sudanese universities: A cross-sectional study
Source: PLoS One. 2022 Jan 26;17(1):e0263067. doi: 10.1371/journal.pone.0263067 (PMC8791537; doi:10.1371/journal.pone.0263067)
Supplement: Attachment — Submitted filename: Response to Reviewer Comments. .pdf [file pone.0263067.s001.pdf]

Reviewer #1:

- The data analysis information written in the abstract is written in tandem with the order present in the data analysis part in the methodology section of the manuscript.

Reviewer #2:

- The questionnaire was pretested and further developed for robustness following a pilot test. The results of the pilot test were used to arrive at the prevalence rate of antibiotic self-medication implemented in the sampling equation used to calculate the sample size in this study.
